# Immunological responses in SARS-CoV-2 and HIV co-infection versus SARS-CoV-2 mono-infection: case report of the interplay between SARS-CoV-2 and HIV

**DOI:** 10.1186/s13223-023-00846-8

**Published:** 2023-10-17

**Authors:** Shima Shahbaz, Wendy Sligl, Mohammed Osman, Shokrollah Elahi

**Affiliations:** 1 School of Dentistry, Division of Foundational Sciences, University of Alberta, Edmonton, AB T6G 2E1 Canada; 2 Department of Critical Care Medicine, University of Alberta, Edmonton, AB T6G 2E1 Canada; 3 Department of Medicine, Division of Infectious Diseases, University of Alberta, Edmonton, AB T6G 2E1 Canada; 4 Department of Medicine, Division of Rheumatology, University of Alberta, Edmonton, AB T6G 2E1 Canada; 5 Department of Oncology, University of Alberta, Edmonton, AB T6G 2E1 Canada; 6 Li Ka Shing Institute of Virology, Faculty of Medicine and Dentistry, University of Alberta, Edmonton, AB T6G 2E1 Canada

**Keywords:** HIV, SARS-CoV-2, COVID-19, Co-infection, ART-naive

## Abstract

**Background:**

There is an urgent need to understand the interplay between SARS-CoV-2 and HIV to inform risk-mitigation approaches for HIV-infected individuals.

**Objectives:**

We conclude that people living with HIV (PLWH) who are antiretroviral therapy (ART) naïve could be at a greater risk of morbidity or mortality once co-infected with SARS-CoV-2.

**Methods:**

Here, we performed extensive immune phenotyping using flow cytometry. Moreover, to compare the range of values observed in the co-infected case, we have included a larger number of mono-infected cases with SARS-CoV-2. We also quantified soluble co-inhibitory/co-stimulatory molecules in the plasma of our patients.

**Results:**

We noted a robust immune activation characterized by the expansion of CD8^+^ T cells expressing co-inhibitory/stimulatory molecules (e.g. PD-1, TIM-3, 2B4, TIGIT, CD39, and ICOS) and activation markers (CD38, CD71, and HLA-DR) in the co-infected case. We further found that neutrophilia was more pronounced at the expense of lymphopenia in the co-infected case. In particular, naïve and central memory CD8^+^ T cells were scarce as a result of switching to effector and effector memory in the co-infected case. CD8^+^ T cell effector functions such as cytokine production (e.g. TNF-α and IFN-γ) and cytolytic molecules expression (granzyme B and perforin) following anti-CD3/CD28 or the Spike peptide pool stimulation were more prominent in the co-infected case versus the mono-infected case. We also observed that SARS-CoV-2 alters T cell exhaustion commonly observed in PLWH.

**Conclusion:**

These findings imply that inadequate immune reconstitution and/or lack of access to ART could dysregulate immune response against SARS-CoV-2 infection, which can result in poor clinical outcomes in PLWH. Our study has implications for prioritizing PLWH in the vaccination program/access to ART in resource-constrained settings.

## Background

The outbreak of the Coronavirus Disease 2019 (COVID-19) has led to an unprecedented global challenge. A better understanding of COVID-19 pathogenesis, in particular, in people with underlying health conditions such as human immunodeficiency virus (HIV) is urgently needed. According to the UNAIDS, 38 million people are living with HIV (PLWH) worldwide, however, only 73% of these individuals are on antiretroviral therapy (ART) [1]. Hence, ~ 10 million PLWH who are not on ART may experience CD4^+^ T cell loss and subsequently compromised adaptive immunity [2]. Because lymphopenia is an indicator of poor clinical outcomes in SARS-CoV-2-infected individuals [3, 4], it is possible to suggest that lymphopenia delays the elimination of SARS-CoV-2, favoring myeloid cell activation and cytokine storm [5–7]. Even though ART suppresses viral replication, HIV-infected individuals experience chronic immune activation characterized by increased pro-inflammatory cytokines, dysregulated regulatory T cell effector functions, and T cell exhaustion [8–10]. Systemic immune activation is associated with comorbid conditions and accelerated aging in PLWH [11, 12]. This immune dysregulation might increase the risk of mortality and morbidity associated with COVID-19. Several epidemiological studies have investigated the clinical features and prognosis of COVID-19 among PLWH with inconsistent results, likely due to disparities in comorbidities [13, 14]. However, recent studies have indicated that untreated advanced HIV (defined as a low CD4^+^ T cell count and lack of ART) are associated with more severe clinical outcomes with SARS-CoV-2 infection [15–17]. Importantly, lower CD4^+^ T cell count has been considered a risk factor for severe COVID-19 irrespective of viral suppression in PLWH [18]. Furthermore, PLWH have a higher rate of comorbidities than the general population, which may contribute to the poor clinical outcomes observed following infection with SARS-CoV-2 [17]. For example, older age, male sex, hypertension, and cardiovascular diseases are associated with an increase in the odds of having severe COVID-19 in PLWH [19]. Similarly, older age, male sex, hypertension, diabetes, tuberculosis, kidney disease, and malignancy are linked to an increase in the likelihood of in-hospital mortality in PLWH [19]. Although the use of ART has been associated with improved clinical outcomes, HIV has remained a risk factor for COVID-19 severity/mortality regardless of viral load [19].

Moreover, it is possible to suggest that residual immune perturbation in PLWH may influence the magnitude and quality of the adaptive immune response to SARS-CoV-2 infection. For instance, a more pronounced CD4^+^ T cell decline or inverted CD4/CD8 ratio is considered a prognostic factor for poor clinical outcomes in PLWH [20]. We and others have reported that CD4^+^ T cells exhibit a more pronounced immune response against SARS-CoV-2 antigens than CD8^+^ T cells [20, 21]. Therefore, CD4^+^ T cell deficiency may influence the humoral immune response as evidenced by a lower IgG conversion rate and a rapid decline in antibody titer in PLWH compared to HIV-uninfected individuals [22].

In addition to antibodies, successful viral clearance requires an effective T cell response [23, 24]. The crucial role of antigen-specific CD8^+^ T cells or cytotoxic T cells (CTLs) in the elimination of virus-infected cells has been well described [23, 25]. However, in the setting of chronic antigenic stimulation (e.g. HIV, HCV, and cancer) CTLs become gradually dysfunctional or exhausted [26]. CTL exhaustion is characterized by the loss of effector functions but upregulation of multiple co-inhibitory receptors (e.g. PD-1, TIM-3) [26, 27]. Although transient up-regulation of co-inhibitory receptors occurs in acute infections, their persistent upregulation is the hallmark of CTL exhaustion [26, 28]. This is particularly relevant for PLWH, in whom the combined effect of lower CD4^+^ T cells/exhausted CTLs and residual immune dysregulation could impair the development of immunity against a new pathogen such as SARS-CoV-2 [29]. To date, limited studies have compared immune responses in HIV/SARS-CoV-2 co-infected versus SARS-CoV-2 mono-infected individuals. To address this knowledge gap, we performed immunophenotyping and functional assays on fresh peripheral blood mononuclear cells from a co-infected patient compared to a SARS-CoV-2 mono-infected individual concurrently admitted to the hospital. Moreover, we have compared the co-infected case with a larger number of mono-infected cases with SARS-CoV-2. Our results reveal robust immune activation in the co-infected case as a potential contributing factor to respiratory failure/pulmonary fibrosis and death.

## Methods

### Study subjects

The diagnosis of COVID-19 infection was performed by RT-PCR specific for viral RNA-dependent RNA polymerase and envelope transcripts (Omicron) using an endotracheal aspirate or nasopharyngeal swab. In addition to our cases, we recruited 64 SARS-CoV-2 infected and ICU-admitted patients for comparison.

### Sample collection and processing

Fresh blood samples were processed and subjected to Ficoll-Hypaque gradients for the isolation of peripheral blood mononuclear cells (PBMCs). PBMCs were cultured in RPMI 1640 (Sigma-Aldrich) supplemented with 10% FBS (Sigma-Aldrich) and 1% penicillin/streptomycin (Sigma-Aldrich).

### Flow cytometry-based immune phenotyping and functional assays

Fluorophore antibodies with specificity to human cell surface antigens and cytokines were purchased mainly from BD Biosciences or Thermo Fisher Scientific. Specifically, the following antibodies were used: anti-CD3 (HIT3a), anti-CD4 (RPA-T4), anti-CD8 (RPA-T8), anti–TIM-3 (7D3), anti–PD-1 (MIH4), anti-CD244 (DM244), anti-ICOS (C398.A4), anti-TIGIT (MBSA43), anti-CD39 (TU66), anti-CD73 (AD2), anti-CCR7 (2-L1-A), anti-CD45RA (HI100), anti-perforin (δG9), anti–granzyme B (GB11), anti–HLA-DR (LN3), anti-CD38 (HIT2),anti-CD71 (MA712), anti-CD235A (HIR2), anti-CD14 (M5E2), anti-CD16 (B73.1), anti-CD33 (WM-53), anti-CD11b (M1/70), anti–TNF-α (MAB11), and anti–IFN-γ (4S.B3). Freshly isolated PBMCs were subjected to immune phenotyping. Similarly, fresh PBMCs were cultured and stimulated with either anti-CD3/CD28 antibodies or SARS-CoV-2 Spike (S) peptide pools (Miltenyi Biotec), which contained the immunodominant sequence domains of the surface glycoproteins S, in the presence of brefeldin A (10 μg/ml) for 6 h according to our previous protocols [8, 30]. Then cells were fixed with 4% paraformaldehyde and acquired on a Fortessa-X20 or LSR Fortessa-SORP flow cytometer (BD Biosciences) and analyzed with FlowJo software (version 10).

### Soluble effector cell checkpoints quantification

We measured soluble levels of checkpoint molecules in the plasma using the U-PLEX effector cell checkpoint combo kit (MSD Meso Scale Diagnostics) [31].

## Results

### Description of Cases

#### SARS-CoV-2 and HIV co-infected case

This was a 40-year-old woman who was admitted to hospital for acute respiratory distress syndrome (ARDS) due to SARS-CoV-2 infection (Omicron variant) for 6 days in March 2022. She was discharged but after 4 days returned to hospital with headache, nausea, cough, chest pain, shortness of breath, and progressive respiratory failure requiring intubation. She was admitted to the intensive care unit (ICU) for mechanical ventilation support. She was also supported with vasopressors for shock. She was treated with dexamethasone and empiric antibiotics. The patient had known untreated HIV infection. She was vaccinated against SARS-CoV-2 but it was unclear whether she had received one or two shots of vaccine. Her CD4^+^ T cell count at the time of admission was 5 cells/mm^3^ (1%) and her most recent plasma viral load count was 150,000 copies/mL. Her complete blood count (CBC) analysis at the time of admission time and 2 days later is summarized in Table 1.

**Table 1:** The complete blood count (CBC) of the co-infected case on the admission day and 2 days later

Test	Result admission day	Abnormality	Result admission 2 days later	Abnormality
WBC (white blood cells)	13.4	High	18.6	High
RBC (red blood cells)	3.66	Low	4.04	
Hemoglobin	90	Low	100	Low
Hematocrit	0.29	Low	0.31	Low
MCV (mean corpuscular volume)	78	Low	78	Low
MCHC (mean corpuscular hemoglobin concentration)	316		318	
RDW (red blood cell distribution Width)	19	High	19.3	High
Platelets	201		227	
Nucleated-RBC	< 1		< 1	
Neutrophils absolute	12.8	High	17.5	High
Immature granulocytes	0.3	High	0.4	High
Lymphocytes	0.1	Low	0.2	Low
Monocytes	0.2		0.4	
Eosinophils	0.0		0.1	
Basophils	0.0		0.0	

In brief, the patient had leukocytosis characterized by a massive expansion of neutrophils and immature granulocytes but lymphopenia. Moreover, she had low red blood cell (RBC), hemoglobin and hematocrit levels and a low mean corpuscular volume (MCV). After readmission to the ICU, which occurred 10 days after the onset of ARDS, a blood sample was drawn for immunological studies.

ART was initiated while in the ICU but despite all the efforts 1 month in ICU, the patient died due to respiratory failure/pulmonary fibrosis and multi-organ failure from COVID-19.

#### SARS-CoV-2 mono-infected case

This was a 49-year-old male with poorly controlled type 2 diabetes mellitus admitted to the ICU with ARDS due to COVID-19 on the same day as the co-infected case. He presented with cough and fever for a few days, and decreased level of consciousness. This patient had received two doses of vaccine in May and June 2021 but no booster. Upon admission to the ICU, he was treated with dexamethasone. He was supported with mechanical ventilation and tracheostomized. CBC on the day of admission and 2 days later showed leukocytosis, characterized by neutrophilia. He was also noted to have low RBC count, hemoglobin, and hematocrit levels (Table 2). The patient improved, was weaned off the ventilator, transferred out of the ICU after 10 days and eventually released from the hospital.

**Table 2:** The complete blood count (CBC) of the mono-infected case on the admission day and 2 days later

Test	Result admission day	Abnormality	Result admission 2 days later	Abnormality
WBC (white blood cells)	11.9	High	10.2	
RBC (red blood cells)	5.56		4.23	Low
Hemoglobin	164		123	Low
Hematocrit	0.47		0.38	Low
MCV (mean corpuscular volume)	85		89	
MCHC (mean corpuscular hemoglobin concentration)	347		327	
RDW (red blood cell distribution Width)	13.6		13.7	
Platelets	241		170	
Nucleated-RBC	< 1		< 1	
Neutrophils absolute	9.8	High	7.4	
Immature granulocytes	0.1		0.1	
Lymphocytes	1.3		1.6	
Monocytes	0.7		1.0	
Eosinophils	0.0		0.1	
Basophils	0.0		0.0	

### A massive expansion of CD8^+^ T cells expressing co-inhibitory/stimulatory receptors in the HIV/SARS-CoV-2 co-infected case versus SARS-CoV-2 mono-infected patient

**Fig. 1:**
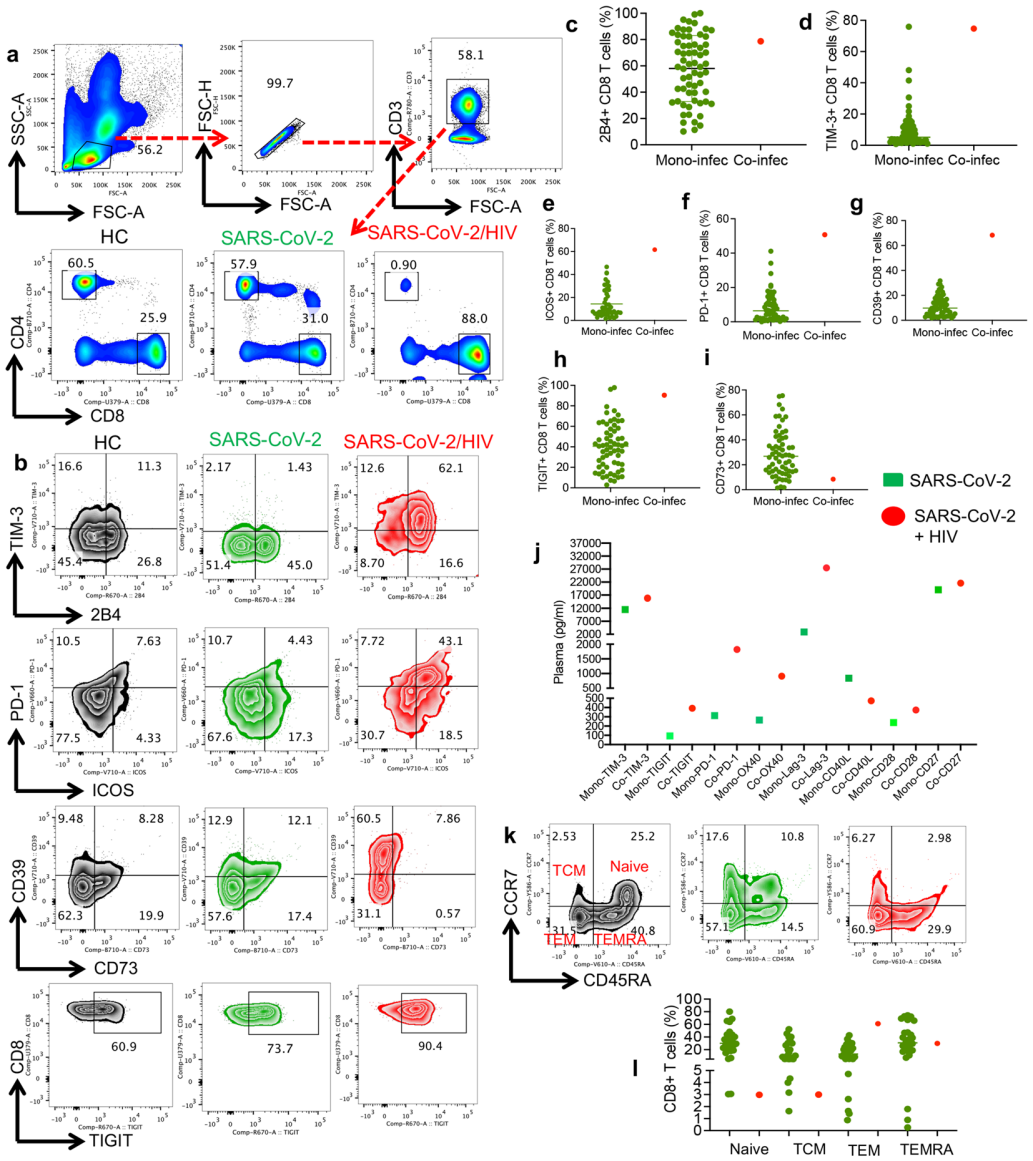
Expansion of CD8^+^ T cells expressing co-inhibitory/stimulatory receptors in the HIV/SARS-CoV-2 co-infected case. **a** Representative flow cytometry plot of the gating strategy and percentages of CD4^+^ and CD8^+^ T cells in the peripheral blood mononuclear cells (PBMCs) of a healthy control (HC), SARS-CoV-2 mono-infected or co-infected (HIV + SARS-CoV-2). **b** Representative flow cytometry plots of TIM-3/2B4, PD-1/ICOS, CD39/CD73, and TIGIT expressing CTLs of mono-and-co-infected individuals and a HC. **c–i** Cumulative data of 2B4^+^, TIM-3^+^, ICOS^+^, PD-1^+^, CD39^+^, TIGIT^+^, and CD73^+^CD8^+^ T cells in ICU admitted mono-infected patients vs. the co-infected case, respectively. **j** The plasma levels of different co-inhibitory and co-stimulatory molecules in the mono-and-co-infected cases. **k** Representative plots, and **l** cumulative data showing the percentages of naïve, T central memory (TCM), T effector memory (TEM), and effector memory re-expressing CD45RA (TEMRA) CD8^+^ T cell in the co-infected case vs. the ICU admitted cohort of mono-infected individuals

To better understand how HIV/SARS-CoV-2 coinfection influences immune cell phenotype and function, we sought to conduct immunophenotyping on these two cases. The co-infected case had extremely low levels of CD4^+^ T cells compared to the SARS-CoV-2 mono-infected patient and a healthy control (HC, male 50-year-old) who was not infected with SARS-CoV-2 but vaccinated twice (Fig. 1a). However, CD8^+^ T cells were enriched in the co-infected case as reported in PLWH [23]. The low frequency of CD4^+^ T cells in the co-infected case made further immunophenotyping of this T cell subset impractical. As such, we decided to characterize CD8^+^ T cells in terms of co-inhibitory receptor expression and activation status. Our analysis revealed substantial expansion of CD8^+^ T cells or cytotoxic T lymphocytes (CTLs) expressing a wide range of co-inhibitory/stimulatory receptors such as TIM-3, 2B4 (CD244), PD-1, ICOS, CD39, and TIGIT in the HIV/SARS-CoV-2 co-infected case compared to the mono-infected case and HC (Fig. 1b). Of note, the frequency of CTLs expressing ICOS, PD-1, CD39, TIM-3, and TIGIT were distinctively higher in the co-infected case than the mono-infected case (Fig. 1b). Moreover, we noted that the proportion of CTLs expressing different co-inhibitory receptors was clearly higher in the co-infected case when compared to 64 ICU-admitted mono-infected individuals with SARS-CoV-2 (Fig. 1c–h). In contrast, CD73 expressing CTLs were diminished in the co-infected case compared to the mono-infected case (Fig. 1b) and the larger ICU-admitted mono-infected cohort (Fig. 1i). Moreover, we quantified the levels of different checkpoint molecules in the plasma of mono- and-co-infected cases. These analyses revealed higher levels of soluble TIM-3, TIGIT, PD-1, OX40, Lag-3, CD28 and CD27 in the plasma of HIV-SARS-CoV-2 co-infected versus the mono-infected case. However, CD40L level was lower in the co-infected versus mono-infected case (Fig. 1j).

### Abundance of highly activated effector and effector memory CTLs in the co-infected case

**Fig. 2:**
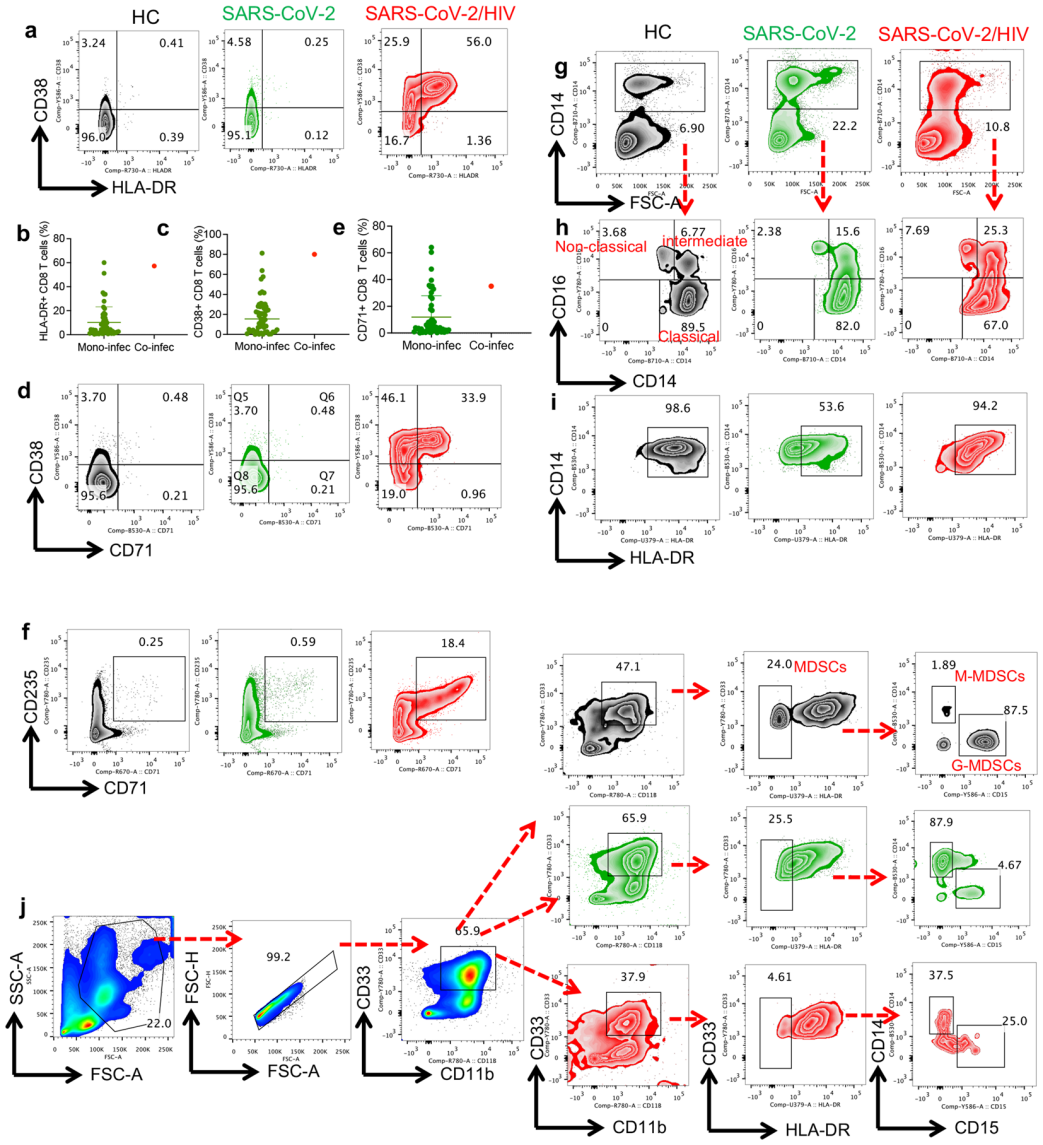
**a** Enhanced CD8^+^ T cells activation, increased the frequency of CECs, and alteration in the frequency of different monocyte subpopulations in the co-infected case. **a** Representative flow cytometry plots, and **b** cumulative data of percentages of CD38/ and **c** HLA-DR expression in CD8^+^ T cells from a HC, mono-and-co-infected individuals. **d** Representative plots, and **e** cumulative data of percentages of CD8^+^CD71^+^ T cells in the mono-infected compared to the co-infected case and a HC. **f** Flow cytometry plots showing percentages of CD71^+^CD235a^+^ cells in PBMCs from a HC, mono-and-co-infected individuals. **g** Flow cytometry plots of percentages of monocytes (CD14 +) in PBMCs from a HC, mono-and-co-infected individuals. **h** Percentages of different monocytes subpopulations (classical, non-classical, and intermediate monocytes) in PBMCs of the mono-infected cohort compared to the co-infected individual and a HC. **i** Flow cytometry plots of percentages of CD14^+^HLADR^+^ monocytes in the mono-infected cohort compared to the co-infected case and a HC. **j** The gating strategy and representative plots of myeloid-derived suppressor cells (MDSCs) and their subpopulations either monocytic (M-MDSC) or granulocytic (G-MDSC) in PBMCs from a HC, mono-and-co-infected individuals

To gain a deeper understanding of the impact of co-infection on CTL phenotype, we analyzed the percentage of different CTL subsets in the co-infected versus mono-infected individuals. Our results revealed a noticeable decline in the frequency of naïve and T central memory (TCM) CD8^+^ T cells but an increased proportion of effector memory (TEM) and effector CD8^+^ T cells expressing CD45RA (TEMRA) (Fig. 1k). This difference was also noticeable when the co-infected individual was compared to the cohort of mono-infected ICU-admitted individuals (Fig. 1l). Moreover, we observed a higher proportion of activated CTLs expressing HLA-DR and CD38 in the co-infected case when compared to the mono-infected case (Fig. 2a) and even to a larger cohort of mono-infected patients with SARS-CoV-2 (Fig. 2b, c). Similarly, we found an increase in the abundance of CD71^+^CTLs in the co-infected case (Fig. 2d, e). This suggests that CTLs in the co-infected case possess a higher proliferative capacity, considering that CD71 is highly co-expressed with Ki67 in activated T cells [32]. Overall, these observations indicate that CTLs in the co-infected case exhibit a highly-activated phenotype compared to their counterparts in the mono-infected case.

### CD71^+^ erythroid cells (CECs) are abundant in the co-infected case

CECs co-express CD71 and CD235a in humans and considered as erythroid progenitors/precursors [33]. It is reported that CECs are expanded in the peripheral blood of SARS-CoV-2-infected individuals, which is more prominent in infected individuals with the Wuhan strain of SARS-CoV-2 than the Delta and Omicron variants [34, 35]. CECs not only have immunosuppressive properties [36] but also get infected with SARS-CoV-2, which may in part explain the mechanism associated with hypoxia in COVID-19 patients [6]. Therefore, we examined the proportion of CECs in the peripheral blood of the mono- and-co-infected cases. We found that the co-infected case had a very high proportion of CECs compared to the mono-infected case and the HC (Fig. 2f).

### Increased frequency of non-classical monocytes but decreased myeloid-derived suppressor cells (MDSCs) in the co-infected case

Immune dysregulation manifested by the expansion of myeloid cells at the expense of lymphopenia is commonly observed in severe COVID-19 [37]. Therefore, we investigated the frequency of monocytes and their subsets including classical (CD14^hi^ CD16^−/lo^), non-classical (CD14^−/lo^ CD16^hi^), and intermediate monocytes (CD14^hi^ CD16^hi^) in the co-infected case compared to the mono-infected individual. The frequency of total monocytes in the co-infected case was 50% lower than the mono-infected individual (Fig. 2g) but the percentage of non-classical monocytes was higher in the co-infected case (Fig. 2h). The proportion of intermediate monocytes remained unchanged in the co-infected case versus the mono-infected case (Fig. 2h). We further characterized monocytes based on the expression of HLA-DR and found that the proportion of HLA-DR expressing monocytes was higher in co-infected versus the mono-infected case (Fig. 2i). Finally, we characterized the frequency of MDSCs in our patients, which revealed a low percentage of MDSCs (4.6%) in the co-infected case compared to the mono-infected individual (25.5%) and HC (24%) (Fig. 2j).

### CTLs from the co-infected case exhibit greater cytokine production and cytolytic molecule expression

**Fig. 3:**
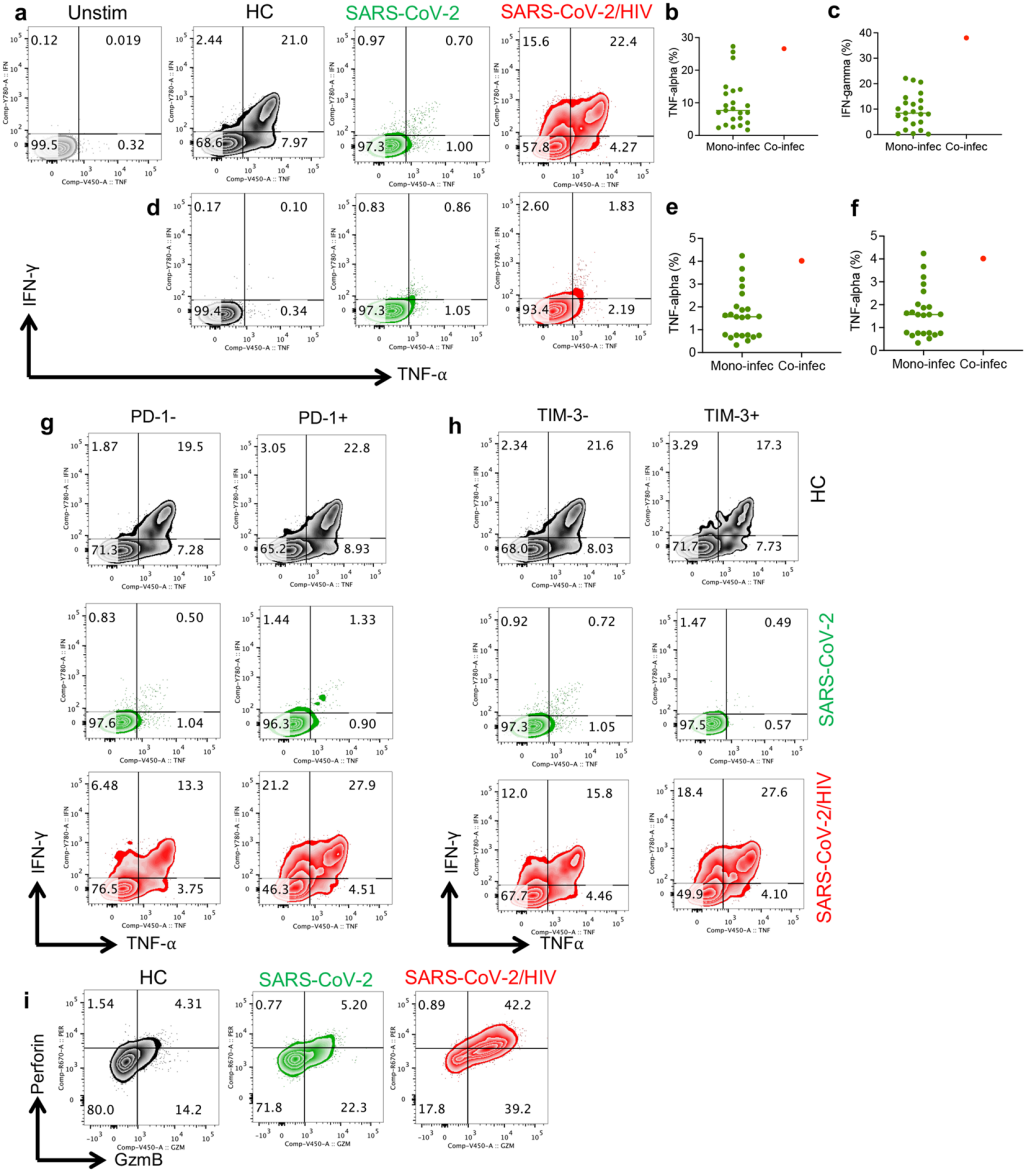
Robust cytokine and cytolytic molecules expression in the co-infected versus mono-infected case. **a** Representative flow cytometry plots, **b** cumulative data of percentages of TNF-α, and **c** IFN-γ and expressing CTLs following stimulation with anti-CD3/CD28 from a HC, mono-and-co-infected individuals. Unstimulated control (Unstim). **d** Representative flow cytometry plots, **e** cumulative data of percentages of TNF-α, and **f** IFN-γ and expression in CTLs following stimulation with the S peptide pools from a HC, mono-and-co-infected individuals. **g** Flow cytometry plots of IFN-γ and TNF-α expression in CD8^+^PD-1^−^ and CD8^+^PD-1^+^ T cells of a HC, mono-and-co-infected individuals following stimulation with anti-CD3/CD28. **h** Flow cytometry plots of IFN-γ and TNF-α expression in CD8^+^TIM-3^−^ and CD8^+^TIM-3^+^ T cells of a HC, mono-and-co-infected individuals following stimulation with anti-CD3/CD28. **i** Flow cytometry plots of percentages of perforin and granzyme B (GzmB) expressing cells among CD8.^+^ T cells of a HC, mono-and-co-infected individuals following stimulation with anti-CD3/CD28

HIV infection is associated with the chronic upregulation of co-inhibitory receptors, which subsequently results in CTL exhaustion [8, 23, 26]. To determine the effector functions of CTLs, we quantified IFN-γ and TNF-α expression following stimulation with anti-CD3/CD28 and the Spike peptide pools of SARS-CoV-2. We observed that CTLs from the co-infected case had more robust IFN-γ and TNF-α expression compared to the mono-infected case (Fig. 3a–c). Similar observations were made when we measured IFN-γ and TNF-α response to stimulation with the S peptide pool of SARS-CoV-2 (Fig. 3d–f).

Moreover, we investigated the effector functions of CTLs in regard to the expression of PD-1 and TIM-3. Although the expression of PD-1 and TIM-3 is associated with CTL exhaustion in chronic conditions such as HIV and cancer [8, 31, 38], this has not been the case in CTLs from SARS-CoV-2 infected individuals [21]. To better delineate the effector functions of PD-1 + and TIM-3 + CTLs compared to their negative counterparts, we analyzed their cytokine production and cytolytic molecule expression. Interestingly, our results showed that TIM-3 and PD-1expressing CTLs exhibited a greater ability to secrete cytokines compared to their negative siblings (Fig. 3g, h). Of note, CTLs did not show an exhausted phenotype in the co-infected case but rather a highly-activated phenotype. Finally, we found that CTLs from the co-infected case had robust granzyme B (GzmB) and perforin expression compared to the mono-infected case and HC (Fig. 3i).

## Discussion and conclusions

Here, we aimed to gain a better understanding of the immune response in an HIV/SARS-CoV-2 co-infected case compared to a SARS-CoV-2 mono-infected individual. Most PLWH who are on ART have an undetectable viral load and reconstituted CD4^+^ T cell count. It is well documented that SARS-CoV-2 infection is associated with lymphopenia [6]. Therefore, SARS-CoV-2 infection imposes a greater risk of lymphopenia in PLWH, in particular, in those who are ART naïve with a lower CD4^+^ T cell count. This was evident in our co-infected case with low absolute whole blood lymphocyte count and < 1% CD4^+^ T cell count in PBMCs. In the absence of ART, uncontrolled viral replication acts as the main driving force for CD4^+^ T cell depletion and immune dysregulation [12]. This was evidenced by the hyper-immune activation characterized by the expansion of CTLs expressing activation markers (CD38/HLA-DR) and co-stimulatory molecules in the co-infected case. This agrees with another study that has reported significantly higher proportion of activated (CD38/HLA-DR) CD4^+^ and CD8^+^ T cells in SARS-CoV-2 co-infected and HIV-unsuppressed compared with HIV suppressed individuals [39]. Moreover, a negative correlation between the frequency of activated (CD38/HLA-DR) CD4^+^/CD8^+^ T cells with absolute CD4 counts was noted in co-infected individuals with unsuppressed HIV replication [39]. Upregulation of co-inhibitory receptors (e.g. PD-1 and TIM-3) in T cells of COVID-19 patients has been documented [6, 40]. Most of these reports speculated that T cells expressing co-inhibitory receptors are exhausted without conducting any functional studies [3, 41]. Upregulation of co-inhibitory receptors does not necessarily mean an exhausted T cell phenotype [42] as T cell exhaustion is characterized by functional impairment of T cell effector functions [26]. Notably, transient expression of co-inhibitory receptors on activated T cells is meant to inhibit a robust and deleterious immune response in an acute setting [43]. The massive production and release of inflammatory cytokines and cytolytic molecules (GzmB and perforin) in T cells expressing PD-1 and TIM-3 in the co-infected case compared to their counterparts lacking these co-inhibitory receptors resemble what occurs during T-cell activation [44]. This agrees with our previous report that antigen-specific CD4^+^ (CD154^+^) and CD8^+^ (CD137^+^) T cells expressing co-inhibitory receptors had significantly higher levels of cytokine (e.g. TNF-α and IFN-γ) production compared to their negative siblings [21]. A similar observation has been reported for antigen-specific PD-1^+^CD4^+^ T cells with greater IFN-γ producing capacity, which supports their activated phenotype [20]. Of note, lower IFN-γ production by T cells in acute COVID-19 patients is widely reported [3, 21, 45]. However, our co-infected case exhibited a robust IFN-γ response to both global and antigen-specific stimulation. These observations indicate that SARS-CoV-2 infection alters exhausted CTLs to an activated phenotype in PLWH. This implies that IFN-γ responses were not impaired in the co-infected case despite the anticipated T cell exhaustion due to untreated HIV infection [8, 23, 27]. Therefore, hyper-T cell activation and a reduced proportion of MDSCs may exacerbate the immune response and contribute to cytokine storm commonly observed in COVID-19 patients [5]. The hyperimmune activation is also accompanied by the expansion of non-classical monocytes in PLWH with inflammatory properties [46]. Similarly, expansion of HLA-DR + monocytes supports their robust activation status [5]. The striking observation was the disappearance of neutrophilia 2 days post ICU admission in the SARS-CoV-2 mono-infected case but a robust expansion of neutrophils (from 12.8 to 17.5 × 10^6^/ml blood) in the co-infected case at the same time. It is reported that neutrophilia is more pronounced in COVID-19 patients with severe or critical disease [47, 48]. In line with this, it was found that among COVID-19 patients’ non-survivors had a greater number of neutrophils than survivors [48, 49]. It is worth mentioning that despite a high viral load and low CD4 count, our co-infected case did not exhibit signs of other infections at the time of admission to the ICU.

Of note, CECs are almost absent or in very low frequency in the peripheral blood of healthy individuals [33, 50]. However, their massive expansion in the peripheral blood of acute COVID-19 patients infected with the SARS-CoV-2 (Wuhan strain) has been reported [6, 34]. It is worth mentioning that those infected with the delta and omicron variants display lower frequency of CECs in their circulation [35]. Similarly, a higher proportion of CECs in the peripheral blood of HIV-infected individuals, in particular, the ART-naïve subpopulation has been detected [51]. However, SARS-CoV-2 variants differentially affect erythropoiesis and the Omicron variant is associated with the least expansion of CECs in the peripheral blood of COVID-19 patients [35]. Therefore, a substantial expansion of CECs in the co-infected case versus mono-infected case highlights the synergistic effects of HIV and SARS-CoV-2 on erythropoiesis dysregulation. This was further demonstrated by the lower number of RBCs and anemia in the co-infected case.

Collectively, our results provide insight on the interplay between SARS-CoV-2 and HIV infection. However, there are inconsistencies regarding the influence of SARS-CoV-2 infection on HIV infection. For instance, it has been reported that co-infection of HIV does not increase disease severity by SARS-CoV-2 [52]. This could be explained by performing such studies in societies where every HIV-infected individual has access to ART rather than low resource setting countries. However, emerging results support a greater risk of morbidity and mortality among HIV/SARS-CoV-2 infected individuals, especially in those with uncontrolled viremia and low CD4 + T cell count [53, 54].

However, there are a few study limitations that should be taken into consideration. This is a single case report, which is a major study limitation. Secondly the co-infected case was a female but the mono-infected case was a male. Hence, sex as a biological factor may influence the results [55]. However, we have provided cumulative data to compare the range of values observed in a larger mono-infected cohort versus the co-infected case. Despite these limitations, we still believe our study provides an insight into the interplay between HIV and SARS-CoV-2. Therefore, the subpopulation of PLWH with insufficient immune reconstitution might be more vulnerable to SARS-CoV-2 infection, the development of COVID-19 disease and its associated complications as reported by others [56]. The coexistence of both viruses in those with lower CD4^+^ T cell count may evolve immune escape mutations leading to adverse public health outcomes. Additionally, unsuppressed HIV replication and hyper immune activation may lead to the depletion of CD4^+^ T cells and subsequently diminished SARS-CoV-2 antigen-specific T cell response [39]. As such, HIV-associated immune dysregulation in viremic individuals may negatively impact vaccine-induced cellular immunity. Therefore, integration of testing strategies or providing rapid SARS-CoV-2 tests to HIV-infected individuals in low-resource setting countries should be considered.

## Data Availability

All data related to this study are incorporated in the text and figures.
